# Intermittent Calf Compression Delays the Onset of Presyncope in Young Healthy Individuals

**DOI:** 10.3389/fphys.2019.01598

**Published:** 2020-01-23

**Authors:** Brooke C. D. Hockin, Victoria E. Claydon

**Affiliations:** ^1^Department of Biomedical Physiology and Kinesiology, Simon Fraser University, Burnaby, BC, Canada; ^2^International Collaboration On Repair Discoveries, The University of British Columbia, Vancouver, BC, Canada

**Keywords:** syncope, compression stockings, cardiovascular, orthostasis, filtration, venous pooling, orthostatic tolerance

## Abstract

Orthostatic fluid shifts reduce the effective circulating volume and thus contribute to syncope susceptibility. Recurrent syncope has a devastating impact on quality of life and is challenging to manage effectively. To blunt orthostatic fluid shifts, static calf compression garments are often prescribed to patients with syncope, but have questionable efficacy. Intermittent calf compression, which mimics the skeletal muscle pump to minimize pooling and filtration, holds promise for the management of syncope. We aimed to evaluate the effectiveness of intermittent calf compression for increasing orthostatic tolerance (OT; time to presyncope). We conducted a randomized single-blind crossover study, in which participants (*n* = 21) underwent three graded 60° head-up-tilt tests to presyncope with combined lower body negative pressure on separate days. Low frequency intermittent calf compression (ICLF; 4 s on and 11 s off) at 0–30 and 0–60 mmHg was applied during two tests and compared to a placebo condition where the garment was fitted, but no compression applied. We measured continuous leg circumference changes (strain gauge plethysmography), cardiovascular responses (finger plethysmography; Finometer Pro), end tidal gases (nasal cannula), and cerebral blood flow velocity (CBFv, transcranial Doppler). The 0–60 mmHg ICLF increased OT (33 ± 2.2 min) compared to both placebo (26 ± 2.4 min; *p* < 0.001) and 0–30 mmHg ICLF (25 ± 2.7 min; *p* < 0.001). Throughout testing 0–60 mmHg ICLF reduced orthostatic fluid shifts compared to both placebo and 0–30 mmHg ICLF (*p* < 0.001), with an associated improvement in stroke volume (*p* < 0.001), allowing blood pressure to be maintained at a reduced heart rate (*p* < 0.001). In addition, CBFv was higher with 0–60 mmHg ICLF than 0–30 mmHg ICLF and placebo (*p* < 0.001). Intermittent calf compression is a promising novel intervention for the management of orthostatic intolerance, which may provide affected individuals renewed independence and improved quality of life.

## Introduction

Syncope, or fainting, refers to a transient loss of consciousness and postural tone due to cerebral hypoperfusion, characterized by a rapid onset, short duration and spontaneous recovery (Shen et al., 2017). Commonly triggered by orthostasis, syncope is a heterogeneous condition caused by inadequate cardiovascular autonomic compensation for the gravitational fluid shifts that occur in the upright position. In patients with orthostatic syncope, impaired autonomic control of vascular resistance and/or cardiac output culminates in global cerebral hypoperfusion (Hansson, 1979; Hainsworth, 2004).

The lifetime cumulative incidence of syncope is estimated to be >35% in the general population, and has a bimodal age distribution, occurring most frequently in adolescent and elderly individuals (Ganzeboom et al., 2006). Syncope accounts for 1–3% of emergency department visits and up to 6% of hospital admissions (Casini-Raggi et al., 2002; McCarthy et al., 2009). Clinical investigations for syncope are largely aimed at the differential diagnosis, ruling out serious or life-threatening conditions such as cardiac syncope, epilepsy, and transient ischemic attacks, with a low diagnostic yield (Mendu et al., 2009; Krahn et al., 2013). The fear of missing a serious underlying condition has led to a practice of over-investigating low-risk patients that is taxing on the healthcare system and causes unnecessary anxiety and uncertainty for patients and families.

Up to 30% of individuals with syncope experience recurrent and severe episodes (Providência et al., 2011), which have a devastating impact on quality of life – associated falls result in injury, while recurrent episodes are distressing and lead to reduced school attendance or community participation, unemployment, and loss of independence (Linzer et al., 1991; Anderson et al., 2012; Sun, 2013). Furthermore, syncope is associated with considerable morbidity; in adults, morbidity is equivalent to patients with chronic low back pain and severe rheumatoid arthritis, while in pediatric populations it is equivalent to patients with asthma, structural heart disease and end-stage renal disease (Linzer et al., 1991; Anderson et al., 2012).

Recurrent syncope is difficult to manage effectively. Patient counseling and lifestyle advice are the primary management recommendations; this includes patient education, advising the patient to avoid known triggers, increase their fluid intake (often with salt supplementation) and to perform physical counter-pressure maneuvers [e.g., squatting, leg crossing, limb and/or abdominal contraction (van Dijk et al., 2006)] when symptoms of presyncope present (Shen et al., 2017). These recommendations are certainly beneficial, but are not usually sufficient to fully prevent symptoms in severely affected patients, particularly those who experience frequent episodes. Furthermore, counter-pressure maneuvers are only useful in patients with a sufficiently long prodrome to employ them, and can be difficult to perform in those with impaired gait or neuromuscular disorders. They are less effective in patients older than 60 years (Brignole et al., 2018). Additional strategies employed in the management of recurrent syncope include pharmacological therapy, aimed at augmenting vascular resistance (Midodrine) or increasing plasma volume (Fludrocortisone), as well as cardiac pacing for syncope with profound cardioinhibition; however, the efficacy and utility of these approaches has been queried (El-Bedawi et al., 1994; Connolly et al., 2003; Salim and Di Sessa, 2005; Maggi and Brignole, 2007).

Orthostatic fluid shifts are a major contributor to syncope susceptibility, and accordingly, present a key therapeutic target for the management of recurrent episodes. Upon standing, there is an immediate gravitational redistribution of the blood volume, with blood accumulating in the veins of the legs and splanchnic circulation due to their increased compliance and capacitance (White and Tsikouris, 2000; Stewart, 2013). Termed “venous pooling,” this fluid redistribution decreases the effective circulating volume, reducing venous return, blood pressure and ultimately, cerebral blood flow (White and Tsikouris, 2000; Ricci et al., 2015). This fluid loss is exacerbated with prolonged standing as the increased venous volume and hydrostatic pressure promotes capillary filtration (Brown and Hainsworth, 1999). In the legs, the accumulated fluid loss is considerable, with 500 ml collecting in a short 10-min head-up tilt (HUT) at 60° (Brown and Hainsworth, 1999). In patients with syncope, this fluid loss is often exaggerated, and is thus an important contributor to the pathophysiology of these disorders (Brown and Hainsworth, 1999).

In an attempt to minimize orthostatic fluid shifts and associated reductions in blood pressure, patients with recurrent syncope are often prescribed static compression garments. These garments apply targeted external counter pressures over the legs and/or splanchnic circulation and are currently a class IIa recommendation for orthostatic syncope (Shen et al., 2017; Brignole et al., 2018). Larger compression garments that envelop the entire leg and/or abdomen have proven to be effective at mitigating orthostatic fluid shifts and improving hemodynamic control during short bouts of orthostatic stress, but due to reported discomfort and difficulty both getting into and removing them, patient compliance is poor (Benkö et al., 2001; Raju et al., 2007). Furthermore, while sitting, there is a reversal of the pressure gradient over the thighs, which may actually exacerbate pooling (Walker and Lamont, 2008). Below knee calf compression stockings are more comfortable and are thus associated with greater patient adherence (Wade et al., 2017); however, their efficacy in reducing orthostatic fluid shifts and improving orthostatic tolerance, or susceptibility to syncope, has been questioned (Protheroe et al., 2011; Hockin et al., 2019).

In a previous study, we showed that intermittent calf compression outperforms commonly prescribed static compression stockings at reducing venous pooling and capillary filtration in the calf, and effectively improves cardiovascular control, during a short (10-min) orthostatic challenge (Hockin et al., 2019). Intermittent compression imitates the squeezing action of the skeletal muscle pump, minimizing venous pooling by pushing blood up past venous one-way valves and improving orthostatic cardiovascular control by increasing venous return and stroke volume. We further determined that the optimal compression paradigm for preventing orthostatic fluid shifts and ameliorating orthostatic cardiovascular control is low frequency (4 s on – 11 s off) compression from 0 to 60 mmHg (Hockin et al., 2019). This promising alternative to static compression garments has the potential to improve the management of syncope, but has not yet been shown to improve orthostatic tolerance.

In this study, we examined the efficacy of our optimal intermittent calf compression paradigm (0–60 mmHg; 4 s on–11 s off) for improving orthostatic tolerance (time to presyncope, or near-fainting) in healthy participants. In our earlier studies, when the intermittent compression was applied at pressures below 0–60 mmHg, there was some blunting of orthostatic fluid shifts, but the magnitude of this effect was not sufficient to improve hemodynamic responses over a 10-min orthostatic challenge (Hockin et al., 2019). However, it is possible that this modest reduction in fluid shifts could delay the onset of presyncope with a more severe orthostatic challenge. Accordingly, we also considered a second, lower pressure paradigm (0–30 mmHg; 4 s on–11 s off), to examine whether any effect of intermittent calf compression on orthostatic tolerance was dose-dependent. In addition to our primary outcome measure of orthostatic tolerance, we also assessed the influence of the intermittent compression paradigms on venous pooling and capillary filtration, and orthostatic cardiovascular control. We hypothesized that intermittent calf compression would: (i) reduce venous pooling and capillary filtration in the calf: (ii) increase stroke volume and cerebral blood flow velocity; and (iii) reduce reliance on tachycardia to support blood pressure, leading to an increased hemodynamic reserve, and ultimately improving orthostatic tolerance by delaying the onset of presyncope during severe orthostatic stress, in a dose-dependent manner.

## Materials and Methods

### Participants

We recruited 21 healthy young adults (12 males; aged 24.3 ± 4.0 years; height: 175.3 ± 7.5 cm; weight: 72.0 ± 7.9 kg; calf circumference: 37.3 ± 2.3 cm) to participate in this study. Prior to testing, all participants completed a brief medical history; all were free of overt cardiovascular and neurological disease. Of the nine women we tested, five females were using oral contraceptives (with testing conducted during the active phase), one female had a hormonal intrauterine device, and three were naturally cycling. In naturally cycling women, we controlled for menstrual phase, ensuring that all three tests were conducted during the same phase of their menstrual cycle (one participant was tested during the follicular phase, two during the luteal phase). One participant was taking Accutane (Isotretinoin), two participants were using anti-histamines for seasonal allergies, and one participant was taking the following medications at a steady dosage: paroxetine, bupropion, respiridone, and dextroamphetamine.

### Study Design

Head-up tilt testing, combined with lower body negative pressure (LBNP) is the gold standard for orthostatic tolerance testing, and can be used to determine orthostatic tolerance with a repeatability of 1.1 ± 0.6 min (Protheroe et al., 2013). In this randomized, single-blind (experimenter blind), placebo-controlled cross-over study, participants underwent three graded, 60° HUT tests to presyncope with combined LBNP on three separate days. Low frequency intermittent calf compression (ICLF: 4 s on and 11 s off) at 0–30 and 0–60 mmHg was applied starting upon initiation of tilting during two tests and compared to a placebo condition in which the garment was fitted, but no compression was applied. The blinded experimenter responsible for terminating the test could not see the compression cuffs (the LBNP box hid the compression cuffs from experimenter view) and the air pressure source was turned on during all three tests so that noise levels were similar during each condition. Within subjects we controlled for time of day, scheduling each of the three experiments for a similar time. We tested 14 participants during the early morning, and 7 participants during the late morning. We advised participants to eat a light breakfast on the morning of testing and asked them to refrain from vigorous exercise, alcohol and caffeine consumption for 12-h prior to testing.

To standardize cuff placement and evaluate whether metrics of calf girth and muscle size influenced the effectiveness of the ICLF interventions, we took anthropometric measurements at the calf using a standard tape measure and skinfold calipers (Slim Guide^TM^, Creative Health Products, Plymouth, MA, United States) on the first visit before testing. The participant’s maximum calf and gaiter circumferences were measured and marked to size for strain gauges and to guide the placement of the intermittent compression cuffs. The calf circumferences of participants ranged from 33 to 41 cm (37.3 ± 2.3 cm). Skinfold measurements were also taken at the widest level of the calf. Muscle cross-sectional area (MCSA) (Fuller et al., 1999; Protheroe et al., 2011) was calculated as:

(1) $$M\,C\,S\,A=\left(\frac{c\,a\,l\,f\,c\,i\,r\,c\,u\,m\,f\,e\,r\,e\,n\,c\,e^{2}}{4\,\pi }\right)\;-\;\left(\frac{calf\;circuference\;\times \;skinfold\;thickness}{2}\right)\;-\;6$$

### Experimental Protocol

**FIGURE 1:**
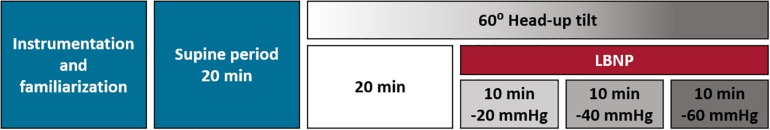
Experimental protocol. On each test day participants provided written informed consent, were familiarized with the experimental procedures, and were instrumented with cardiovascular and plethysmographic monitoring. After 20 min of supine rest they underwent a 60° head-up tilt test to presyncope with combined, graded lower body negative pressure. After 20 min upright, lower body negative pressure was applied and incremented (–20, –40, –60 mmHg) at 10 min intervals. The test was terminated at presyncope, at participant request or upon completion of the entire protocol. Orthostatic tolerance was determined as the time from tilt start to test termination. On each of the three test days, a different compression paradigm was applied: 0–30 mmHg low frequency intermittent compression, 0–60 mmHg low frequency intermittent compression, or a placebo condition where the garment was fitted but no compression was applied. LBNP, lower body negative pressure.

The experimental protocol is shown in Figure 1. While participants laid supine on a manual tilt table with a footboard support, they were instrumented with cardiovascular monitoring equipment and familiarized with the experimental protocol. Participants were given a 20 min supine rest period to assess supine (baseline) cardiovascular parameters and allow pooled fluid in the legs to be reabsorbed. The orthostatic stress test began with 20 min of 60° HUT, after which, LBNP was applied and incremented (−20, −40, −60 mmHg) at 10 min intervals while the participant was still upright. The test was terminated at presyncope, at participant request, or upon completion of the entire protocol. Presyncope was defined as a systolic blood pressure less than 80 mmHg, or a heart rate (HR) below 50 bpm or greater than 170 bpm, and/or the onset of presyncopal symptoms such as nausea, light-headedness, tunnel-vision, warmth and perspiration. The manual tilt table was rapidly tilted back to the supine position when any of these end-points were reached, orthostatic tolerance was defined as the time in minutes from tilt start to test termination (the time to presyncope).

### Cardiovascular Monitoring

We used the Finometer Pro^TM^ device (Finometer, Finapres Medical Systems, Amsterdam, Netherlands) to monitor cardiovascular parameters continuously and non-invasively throughout testing. This device measures beat-to-beat systolic (SAP), diastolic (DAP) and mean (MAP) arterial pressure from the digital arteries in the middle finger using photoplethysmography and reconstructs the brachial arterial pressure waveform. This device is equipped with the Modelflow algorithm to enable estimation of stroke volume (SV) on a beat-to-beat basis (Wesseling et al., 1993; Jellema et al., 1996; Harms et al., 1999). A lead II electrocardiogram (ECG; Finapres ECG Module, Finapres Medical Systems, Amsterdam, Netherlands) monitored HR and rhythm throughout testing and cardiac output (CO) was calculated as the product of HR and SV. Total peripheral resistance (TPR) was calculated as MAP divided by CO. We also measured the partial pressures of end tidal oxygen (P_ET_O_2_) and carbon dioxide (P_ET_CO_2_) on a breath-by breath basis (O_2_Cap Oxygen Analyzer, Oxigraf Inc, Sunnyvale, CA, United States) using a nasal cannula. We determined mean cerebral blood flow velocity (CBFv) continuously and non-invasively from the middle cerebral artery using a 2 MHz ultrasound probe positioned over the left temporal window and secured in position using a headband. With the arm supported at heart level, brachial blood flow velocity was recorded continuously and non-invasively using an 8 MHz ultrasound probe, held in place by an adjustable clamp (Doppler Box, Compumedics Germany GmbH, The DWL Doppler Company, Singen, Germany). Forearm vascular resistance (FVR) was calculated as MAP divided by brachial blood flow velocity (Claydon et al., 2019). All cardiovascular recordings were sampled at 1 KHz using an analog-to-digital converter (Powerlab 16/30, AD Instruments, Colorado Springs, CO, United States) and stored for offline analysis.

### Lower Limb Plethysmography

Estimations of limb volume changes (mL/100 mL of tissue), expressed as percentage changes from the supine volume, were performed using strain-gauge plethysmography. We placed two strain gauges on each leg, the first at the level of maximum calf circumference (the compression garment was centered over this strain gauge), while the second was positioned at the gaiter (mid-point between the level of maximum calf circumference and the medial malleolus). The strain gauge positioned at the gaiter was used to monitor the formation of edema distal to the site of compression. Strain gauge plethysmography data were analyzed during the compression-free (off) phase during intermittent compression paradigms, or the equivalent time point during the placebo condition.

### Compression System

**FIGURE 2:**
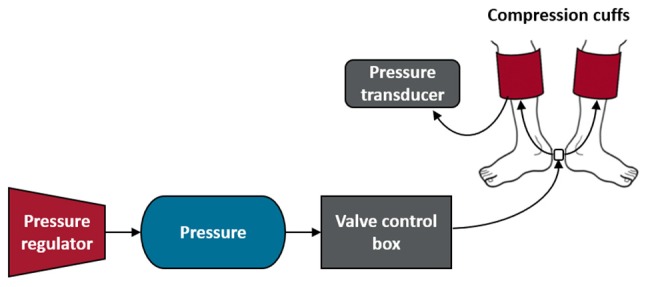
Compression system. The compression system encompassed a pressure regulator, a pressure source, a valve control box and two compression cuffs (adult large), shown above from left to right. The pressure source was connected to a valve control box with an Arduino board and programmable Arduino chip, which allowed us to program the valve system to cycle from open for 4 s to closed for 11 s throughout the test. In addition, the pressure inside one of the compression cuffs was continuously monitored throughout testing with the use of a pressure transducer.

Our custom-made compression system (Figure 2) utilized rapid inflation and deflation rates (<1 s) to deliver the intermittent compression intervention at pressures of 0–30 and 0–60 mmHg. We attached a pressure regulator to a pressure source that allowed us to control the pressure in the cuff. Throughout testing, a pressure transducer (Reusable BP Transducer MLT0380/D, ADInstruments, Colorado Springs, CO, United States) was used to monitor compression pressures within the inflatable compression garments and confirmed that target pressures were successfully applied.

### Data and Statistical Analyses

Analyses were performed by investigators blinded to the test condition. Cardiovascular data are represented as mean values over the final 30 s of every 2 min period for the duration of each tilt test. Calf circumference data derived from strain gauges are reported as percent changes from supine and are similarly presented as 30 s means [during the compression-free (off) phase during intermittent compression paradigms, or the equivalent time point during the placebo condition] at 2 min intervals throughout each tilt. Strain gauge data from the level of maximum calf circumference and gaiter region were averaged for the right and left legs. Supine values (time point zero) for both cardiovascular and strain gauge data were calculated as mean values over the final 30 s of the final minute of the supine period. Absolute FVR data are influenced by the angle of insonation of the brachial ultrasound probe, which could not be standardized between participants, therefore these data are reported only as the percent change from baseline. However, the angle of insonation was maintained within each participant, so evaluations of the percent changes in FVR within the protocol are valid. In theory, measures of FVR would be unreliable if the diameter of the insonated vessel changed during testing. However, we previously confirmed that there is no change in brachial arterial diameter with this level of orthostatic stress, because resistance responses occur in downstream arterioles, and accordingly that changes in velocity are proportional to flow (Claydon et al., 2019). We used nasal sampling to measure P_ET_CO_2_ because it is more comfortable and allows the participant to communicate symptoms throughout testing. However, one caveat of nasal sampling is that some mixing with room air can occur, depending on the placement of the nasal prong, resulting in slightly lower P_ET_CO_2_ values (∼3–5 mmHg), although the changes over time are consistent within a placement position. Accordingly, P_ET_CO_2_ values are also reported only as the change from baseline.

Statistical analyses and data visualization were performed using JMP^®^ (Version 14.1, SAS Institute Inc., Cary, NC, United States) and SigmaPlot (Version 14 Systat Software, Inc, San Jose, CA, United States). We tested data for normality using the Shapiro–Wilk test and data are reported as means ± SEM. Differences were determined to be statistically significant where *p* < 0.05. For the orthostatic tolerance outcomes, we performed a linear mixed model analysis with a Tukey HSD *post hoc* test, a Kaplan–Meier time-to-event Analysis (Gehan–Breslow test), as well as a paired *t*-test on the change in orthostatic tolerance between ICLF conditions and placebo data. To test whether intervention order had an effect on orthostatic tolerance, we ran a linear mixed model analyses with compression condition and intervention order as factors. For cardiovascular and venous pooling data, comparisons between tilt conditions and over time were conducted using linear mixed model analyses with a Tukey HSD *post hoc* test.

We performed correlations between changes in venous pooling and capillary filtration during orthostatic stress with baseline calf circumference or MCSA using the Pearson correlation coefficient.

## Results

Of our 21 participants, 18 were included in the analysis of orthostatic tolerance data. To ensure a complete repeated measures analysis, we excluded individuals who were missing a tilt endpoint in one of the three conditions. One individual was excluded due to technical difficulties with the ICLF in the 0–30 mmHg condition, one individual elected to stop the test early prior to reaching endpoint criteria in the 0–60 mmHg condition, and one individual chose not to return for the 3rd test (0–60 mmHg condition). All 21 individuals were included in secondary analyses of cardiovascular and plethysmographic data.

### Orthostatic Tolerance

**FIGURE 3:**
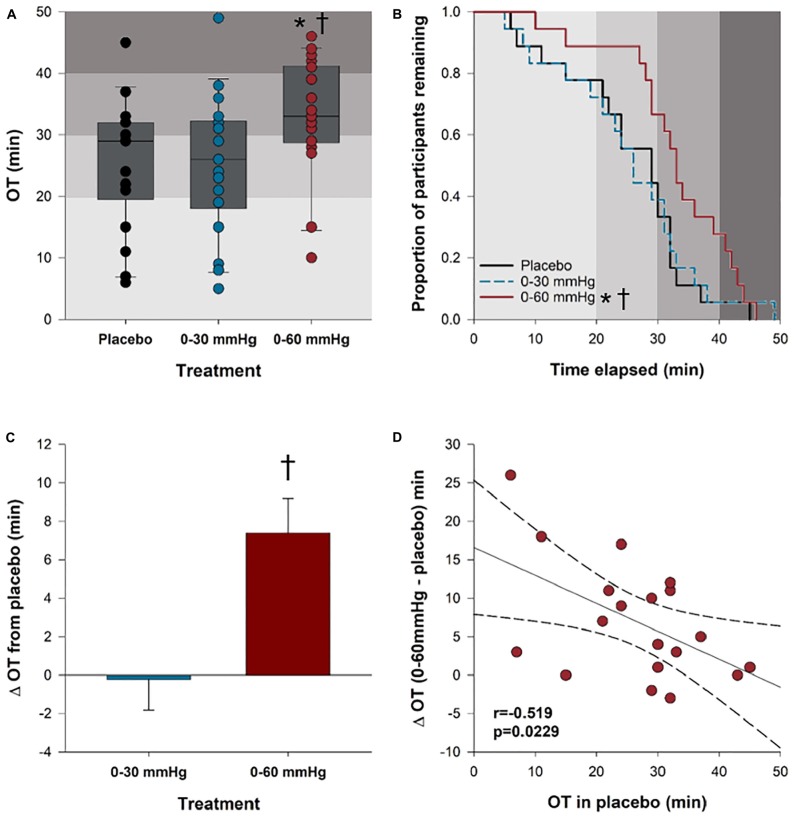
Orthostatic tolerance. **(A)** Orthostatic tolerance, rounded to the nearest minute, is shown for all three tilt conditions. **(B)** Kaplan–Meier plot depicting the proportion of participants remaining (who have not reached presyncope) in each condition at any given tilt time. **(C)** Bar plots showing the mean improvement in orthostatic tolerance for each compression condition relative to the placebo. **(D)** The mean improvement in orthostatic tolerance with 0–60 mmHg compression compared to placebo was dependent on baseline orthostatic tolerance (placebo). ^∗^Significantly different from placebo (*p* < 0.05); **†**significantly different from 0 to 30 mmHg compression (*p* < 0.05). Shaded gray boxes of increasing intensity represent stages of the tilt test and increasing orthostatic stress. OT, orthostatic tolerance.

Orthostatic tolerance results are shown in Figure 3. Orthostatic tolerance (time to presyncope) was significantly improved with 0–60 mmHg (33 ± 2.2 min) ICLF, compared to both the placebo (26 ± 2.4 min; *p* = 0.0009) and 0–30 mmHg (25 ± 2.7 min; *p* = 0.0007) conditions; however, 0–30 mmHg ICLF showed no benefit over the placebo condition (*p* = 0.9921) (Figure 3A). There was no effect of intervention order on orthostatic tolerance (*p* = 0.763) and no interaction between compression condition and intervention order (*p* = 0.977). Kaplan–Meier time-to-event analyses (Figure 3B) similarly showed that time to presyncope was significantly delayed in the 0–60 mmHg condition compared to both the placebo (*p* = 0.0388) and 0–30 mmHg (*p* = 0.0442) conditions. The mean improvement in orthostatic tolerance from placebo with 0–60 mmHg ICLF was +7.4 ± 1.8 min; this was significantly greater than the mean change from placebo with 0–30 mmHg ICLF (−0.2 ± 1.6 min; *p* < 0.001; Figure 3C). The mean improvement in orthostatic tolerance was dependent on orthostatic tolerance in the placebo condition (*r* = −0.519; *p* = 0.0229), such that individuals with a lower baseline or placebo orthostatic tolerance experienced a greater improvement with 0–60 mmHg ICLF (Figure 3D). One caveat of this result is that tests in the 0–60 mmHg condition were more likely to be terminated at participant request due to symptoms and discomfort associated with the high levels of orthostatic stress or due to orthostatic tachycardia, rather than a presyncopal blood pressure drop. In the placebo condition 86% of tilts were terminated because of presyncope (vasovagal response), versus 85% with 0–30 mmHg ICLF and 60% with 0–60 mmHg ICLF (χ^2^: *p* = 0.043). In both the placebo and 0–30 mmHg ICLF conditions 5% of tilts were terminated due to orthostatic tachycardia with symptoms, compared to 10% of tilts in the 0–60 mmHg ICLF condition. With 0–60 mmHg ICLF 30% of tilts were terminated at participant request due to orthostatic symptoms alone, versus 10% of tilts in each of the placebo and 0–30 mmHg ICLF conditions. MCSA was not related to the magnitude of improvement in orthostatic tolerance in either ICLF condition (0–30 mmHg: *r* = −0.265, *p* = 0.258; 0–60 mmHg: *r* = −0.318, *p* = 0.184).

### Venous Pooling and Filtration

**FIGURE 4:**
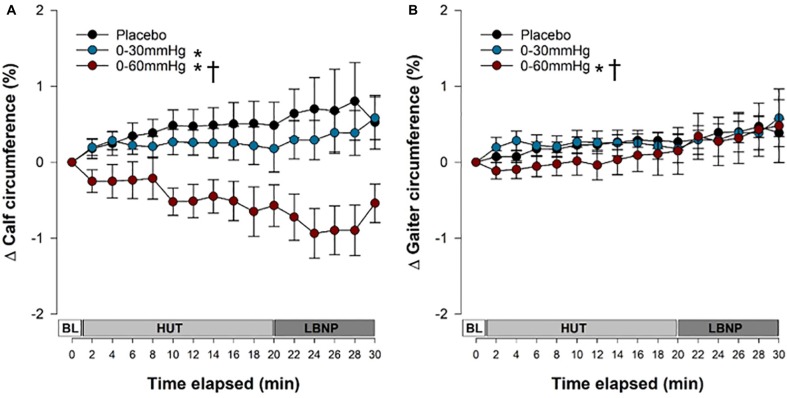
Venous pooling and capillary filtration during tilt testing. The time course of pooling and filtration in the calf **(A)** and at the level of the gaiter **(B)**. Data are presented as a percentage change form supine and symbols (mean ± SEM) reflect mean data from the right and left calves/gaiters combined, from the final 30 s of every 2 min interval. Main effects of condition throughout tilt are shown. ^∗^Significantly different from placebo (*p* < 0.001); **^†^**Significantly different from 0 to 30 mmHg compression. BL, baseline; HUT, head-upright tilt; LBNP, lower body negative pressure.

Changes in calf and gaiter circumference over the course of the tilt test are shown in Figure 4. Data are shown up until the end of the first phase of LBNP, because in the placebo and 0–30 mmHg ICLF conditions only one person was able to tolerate the subsequent phase(s). Both the 0–30 mmHg (*p* = 0.0005) and 0–60 mmHg (*p* < 0.0001) ICLF conditions significantly reduced pooling and filtration at the level of the calf compared to placebo; however, the 0–60 mmHg ICLF additionally outperformed 0–30 mmHg ICLF (*p* < 0.0001). After 20 min of HUT, calf circumference increased by +0.48 ± 0.30% in the placebo condition, which was significantly greater than 0–60 mmHg ICLF (−0.57 ± 0.27%; *p* = 0.0162), but was not different from 0–30 mmHg ICLF (+0.17 ± 0.31%).

At the level of the gaiter, edema distal to the site of compression was not exacerbated with ICLF and, in fact, was reduced. With 0–60 mmHg ICLF gaiter circumference was reduced compared to both 0–30 mmHg ICLF (*p* < 0.0001) and placebo (*p* = 0.0058). With 0–30 mmHg ICLF, gaiter circumference was not different from placebo (*p* = 0.1667). There was a main effect of tilt time elapsed on pooling and filtration at the level of the gaiter (*p* < 0.0001), with pooling and filtration increasing over the course of tilt. After 20 min of HUT, gaiter circumference increased by +0.27 ± 0.11% in the placebo condition, by +0.33 ± 0.19% with 0–30 mmHg ICLF, and by +0.15 ± 0.31% with 0–60 mmHg ICLF compared to supine baseline; these were not statistically significant.

Anthropometric calf circumference measurements and calculated MCSA were not related to the magnitude of pooling at the level of the calf or the gaiter after 20 min of HUT in any of the three conditions.

### Cardiovascular Responses

**FIGURE 5:**
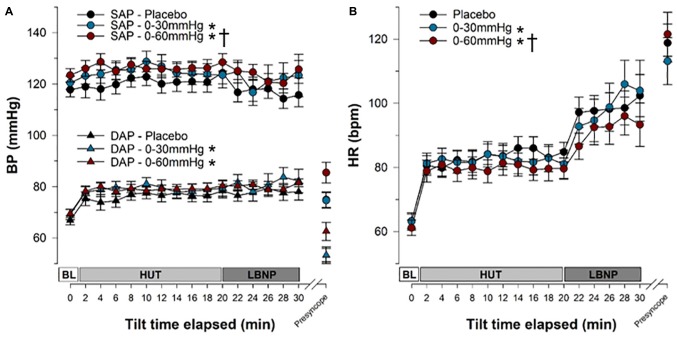
Blood pressure and heart rate responses during tilt testing. The time course of blood pressure **(A)** and heart rate **(B)** during tilt are shown. Symbols (mean ± SEM) reflect averaged data, over the last 30 s of every 2 min interval. Main effects of condition throughout tilt are shown. ^∗^Significantly different from placebo (*p* < 0.001); **^†^**Significantly different from 0 to 30 mmHg compression. BP, blood pressure; SAP, systolic arterial pressure; DAP, diastolic arterial pressure; HR, heart rate; BL, baseline; HUT, head-upright tilt; LBNP, lower body negative pressure.

**FIGURE 6:**
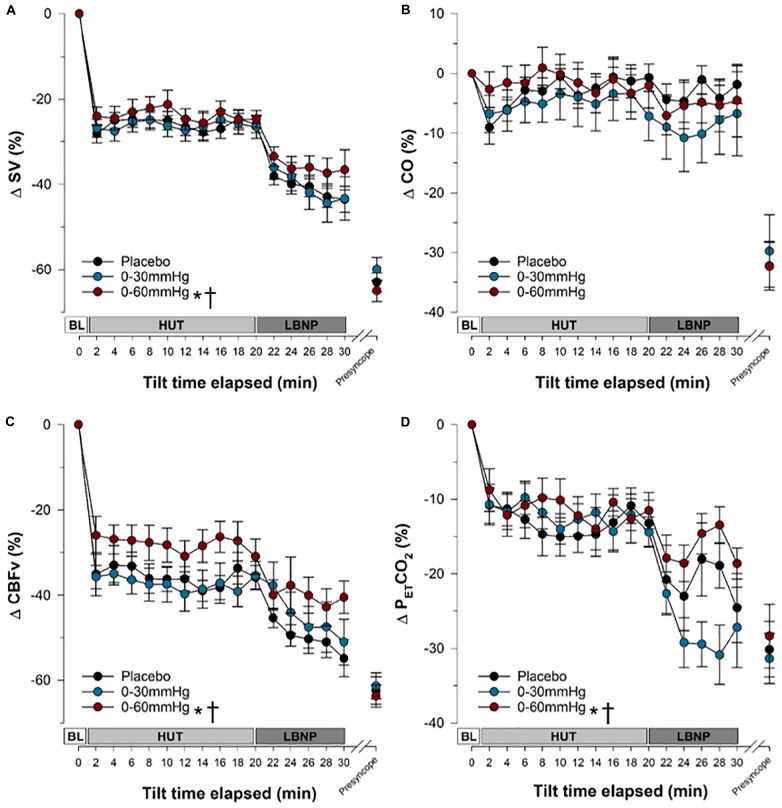
Stroke volume, cardiac output, cerebral blood flow velocity and end-tidal CO_2_ responses during tilt testing. The time course of stroke volume **(A)**, cardiac output **(B)**, cerebral blood flow velocity **(C)** and end-tidal carbon dioxide **(D)** during tilt are shown. Symbols (mean ± SEM) reflect averaged data, over the last 30 s of every 2 min interval and are reported as a percentage change from supine values. Main effects of condition throughout tilt are shown. ^∗^Significantly different from placebo (*p* < 0.001); **^†^**significantly different from 0 to 30 mmHg compression. SV, stroke volume; CO, cardiac output; CBFv, cerebral blood flow velocity; P_ET_CO_2,_ end tidal partial pressure of carbon dioxide; BL, baseline; HUT, head-upright tilt; LBNP, lower body negative pressure.

Supine cardiovascular parameters were similar between compression conditions (Table 1). The time course of cardiovascular responses throughout tilt in the three compression conditions are shown in Figures 5, 6, with main effects of condition indicated. Data are shown up until the end of the first phase of LBNP, because in the placebo and 0–30 mmHg ICLF conditions only one person was able to tolerate the subsequent phase(s). The absolute and percent changes from baseline values for each parameter are shown in Table 1 for key stages of the test: baseline, after 10 min of tilting (Tilt_10_), after 20 min of tilting (Tilt_20_), at the end of the first phase of LBNP (LBNP_30_), and at the presyncope time point, with effects of condition and time indicated. Note that for percent changes, differences between these stages and baseline values are not represented, as the baseline is without variance (0.00 ± 0.00%).

**TABLE 1:** Cardiovascular responses at selected tilt intervals.

		**Baseline**	**Tilt_10_**	**Δ(%)**	**Tilt_20_**	**Δ(%)**	**LBNP_30_**	**Δ(%)**	**Presyncope**	**Δ(%)**
SAP (mmHg)	Placebo	117.8 ± 2.8	122.8 ± 3.4	2.8 ± 2.0	124.3 ± 4.2	4.4 ± 2.8	115.7 ± 4.6	−5.0 ± 3.5	74.9 ± 3.1^∗†‡Ω^	−36.4 ± 2.1^†‡Ω^
	0–30 mmHg	120.5 ± 2.5	128.8 ± 4.0	5.0 ± 2.3	123.5 ± 5.1	5.0 ± 2.3	123.4 ± 8.3	−0.5 ± 5.1	74.7 ± 3.0^∗†‡Ω^	−38.1 ± 2.1^†‡Ω^
	0–60 mmHg	123.4 ± 2.6	125.9 ± 2.9	2.2 ± 1.9	128.5 ± 3.4	3.5 ± 2.2	125.7 ± 3.4	−1.8 ± 3.0	85.4 ± 4.0^∗†‡Ω^	−30.5 ± 3.3^†‡Ω^
DAP (mmHg)	Placebo	66.9 ± 1.8	77.6 ± 1.8^∗^	13.7 ± 2.4	78.3 ± 2.6^∗^	15.3 ± 2.8	78.0 ± 3.2	11.7 ± 2.9	53.3 ± 2.6^∗†‡Ω^	−20.3 ± 3.1^†‡Ω^
	0–30 mmHg	69.2 ± 2.0	81.1 ± 2.4^∗^	15.0 ± 1.6	79.4 ± 2.4	11.3 ± 2.4	82.2 ± 4.5	15.0 ± 3.8	53.3 ± 3.2^∗†‡Ω^	−23.0 ± 2.1^†‡Ω^
	0–60 mmHg	69.5 ± 1.6	78.4 ± 1.7	13.2 ± 2.1	80.3 ± 1.9^∗^	14.8 ± 2.1	81.5 ± 1.6	13.8 ± 2.9	62.6 ± 3.5^†‡Ω^	10.2 ± 4.2^†‡Ωβ^
HR (bpm)	Placebo	63.3 ± 2.3	84.1 ± 3.2^∗^	34.2 ± 2.8	84.8 ± 3.1^∗^	35.7 ± 3.9	102.4 ± 6.6^∗^†‡	77.9 ± 9.2^†‡^	118.8 ± 5.9^∗†‡^	89.9 ± 10.2^†‡^
	0–30 mmHg	63.2 ± 2.7	84.1 ± 4.0^∗^	34.3 ± 4.4	81.0 ± 4.4	27.9 ± 3.8	103.9 ± 9.5^∗‡^	67.6 ± 11.5^‡^	113.1 ± 7.3^∗†‡^	80.2 ± 10.6^†‡^
	0–60 mmHg	61.2 ± 2.4	78.8 ± 3.6^∗^	28.8 ± 3.4	80.0 ± 3.3^∗^	30.4 ± 3.4	93.3 ± 6.8^∗^	54.2 ± 5.5	121.5 ± 6.9^∗†‡Ω^	98.5 ± 10.3^†‡Ω^
SV (mL)	Placebo	90.7 ± 3.6	69.6 ± 3.8^∗^	−25.0 ± 2.1	70.0 ± 3.6^∗^	−25.9 ± 2.0	54.9 ± 5.0^∗^†‡	−43.5 ± 3.1^†‡^	33.0 ± 2.0^∗†‡Ω^	−63.0 ± 2.2^†‡Ω^
	0–30 mmHg	89.3 ± 4.3	68.6 ± 4.7^∗^	−26.3 ± 2.5	68.1 ± 5.0^∗^	−26.5 ± 2.7	54.3 ± 8.2^∗^	−43.3 ± 5.1^†‡^	35.0 ± 2.7^∗†‡^	−59.9 ± 2.8^†‡Ω^
	0–60 mmHg	98.5 ± 4.0	78.4 ± 5.2^∗^	−21.3 ± 3.3	75.3 ± 4.4^∗^	−24.7 ± 2.0	66.4 ± 7.4^∗^	−36.6 ± 4.7^†‡^	34.5 ± 2.7^∗†‡Ω^	−65.0 ± 2.5^†‡Ω^
CO (L^∗^min^–1^)	Placebo	5.72 ± 0.30	5.76 ± 0.32	−0.52 ± 2.0	5.86 ± 0.33	−0.7 ± 2.3	5.39 ± 0.33	−1.86 ± 3.2	3.84 ± 0.26^∗†‡Ω^	−32.2 ± 4.1^†‡Ω^
	0–30 mmHg	5.63 ± 0.33	5.55 ± 0.35	−3.4 ± 4.3	5.29 ± 0.38	−7.2 ± 4.1	5.19 ± 0.45	−6.7 ± 7.1	3.76 ± 0.26^∗†‡^	−29.8 ± 6.1^†‡Ω^
	0–6 0mmHg	6.00 ± 0.32	5.93 ± 0.32	−0.11 ± 3.4	5.84 ± 0.32	−2.1 ± 3.7	5.72 ± 0.45	−4.5 ± 6.1	4.09 ± 0.32^∗†‡Ω^	−32.3 ± 4.0^†‡Ω^
TPR	Placebo	15.5 ± 0.8	17.2 ± 1.1	10.0 ± 3.1	17.0 ± 1.2	10.0 ± 3.1	17.6 ± 1.3	8.0 ± 5.6	17.8 ± 1.1	18.6 ± 6.7
(mmHg^∗^min^∗^L^–1^)	0–30 mmHg	16.4 ± 0.9	18.8 ± 1.3	17.0 ± 5.6	19.2 ± 1.5	17.8 ± 6.3	19.9 ± 2.0	17.8 ± 6.3	18.1 ± 1.5	17.5 ± 14.4
	0–60 mmHg	16.2 ± 1.1	16.8 ± 0.9	7.5 ± 4.5	17.6 ± 1.0	12.8 ± 6.0	18.3 ± 1.4	17.2 ± 7.6	19.5 ± 1.7	25.0 ± 9.5
FVR	Placebo	–	–	82.3 ± 27.4	–	90.5 ± 27.0	–	152.9 ± 54.2	–	72.1 ± 23.3
(mmHg^∗^min^∗^L^–1^)	0–30 mmHg	–	–	122.6 ± 35.9	–	63.6 ± 20.2	–	118.5 ± 35.7	–	59.1 ± 25.5^‡^
	0–60 mmHg	–	–	83.5 ± 18.4	–	72.4 ± 13.6	–	99.0 ± 32.2	–	210.0 ± 50.6^†‡αβ^
CBFv (cm^∗^s^–1^)	Placebo	61.0 ± 4.7	39.9 ± 4.4^∗^	−36.3 ± 2.8	40.2 ± 4.3^∗^	−35.8 ± 2.8	30.5 ± 6.1^∗^	−54.8 ± 4.3^†‡^	22.5 ± 2.4^∗†‡^	−62.4 ± 3.2^†‡^
	0–30 mmHg	61.8 ± 5.3	39.1 ± 4.9^∗^	−37.4 ± 4.2	41.6 ± 5.7^∗^	−35.4 ± 3.3	27.4 ± 4.1^∗^	−51.0 ± 5.3	25.0 ± 3.2^∗†‡^	−61.4 ± 3.1^†‡^
	0–60 mmHg	64.2 ± 6.2	44.7 ± 4.7^∗^	−28.3 ± 4.0	41.8 ± 3.6^∗^	−31.0 ± 4.1	37.0 ± 4.8^∗^	−40.5 ± 3.9^†^	22.3 ± 3.1^∗†‡^	−63.7 ± 2.6^†‡Ω^
P_ET_CO_2_ (mmHg)	Placebo	–	–	−14.2 ± 2.6	–	−12.2 ± 3.2	–	−20.8 ± 3.0	–	−29.4 ± 3.5^†‡^
	0–30 mmHg	–	–	−14.0 ± 2.0	–	−14.4 ± 2.0	–	−27.2 ± 5.4	–	−31.4 ± 3.4^†‡^
	0–60 mmHg	–	–	−10.2 ± 2.8	–	−11.5 ± 2.4	–	−18.6 ± 2.1	–	−28.1 ± 4.0^†‡^

*Data are presented as the mean ± SEM for each condition with percent changes from baseline indicated in shaded columns. Main effects of condition are not shown. Note that for percent changes, statistical differences relative to baseline values are not represented, as the baseline is without variance (0.00 ± 0.00%). Symbols: ^∗^Significantly different from baseline value for given condition; ^†^Significantly different from 10 min time point for given condition (Tilt_10_). ^‡^Significantly different from 20 min time point for given condition (Tilt_20_). ^Ω^Significantly different from the end of the first phase of LBNP for given condition (LBNP_30_). SAP, systolic arterial pressure; DAP, diastolic arterial pressure; HR, heart rate; SV, stroke volume; CO, cardiac output; TPR, total peripheral resistance; FVR, forearm vascular resistance. ^α^Significantly different from placebo condition at given time point. ^β^Significantly different from 0 to 30 mmHg compression condition at given time point.*

Overall, SAP (Figure 5A and Table 1) was significantly increased during tilt with both 0–30 mmHg (*p* = 0.0001) and 0–60 mmHg (*p* < 0.0001) ICLF compared to placebo, and with 0–60 mmHg ICLF compared to 0–30 mmHg ICLF (*p* = 0.0348). When data were expressed as a percent change from baseline, SAP was only increased with 0-60 mmHg ICLF compared to placebo (*p* = 0.0421). At presyncope, SAP was not significantly different between conditions, although it was reduced compared to baseline, Tilt_10_, Tilt_20_, and LBNP_30_ time points in all three conditions (*p* < 0.0001). Throughout tilt, DAP (Figure 5A and Table 1) was significantly higher in the 0–60 and 0–30 mmHg ICLF conditions compared to placebo (*p* < 0001). When data were expressed as a percentage change from baseline, DAP was increased with 0–60 mmHg ICLF compared to 0–30 mmHg ICLF (*p* = 0.0496), but ICLF conditions were not different from placebo. In all three conditions, DAP tended to increase during the tilt test compared to baseline. At presyncope, DAP was reduced compared to baseline, Tilt_10_, Tilt_20_, and LBNP_30_ time points in the placebo and 0–30 mmHg ICLF conditions (*p* < 0.0001). With 0–60 mmHg ICLF DAP was reduced at presyncope compared to Tilt_10_, Tilt_20_, and LBNP_30_ time points (*p* < 0.0001), but was not different from baseline (*p* = 0.3780). DAP tended to be higher at presyncope in the 0–60 mmHg condition compared to placebo (*p* = 0.0577) and 0–30 mmHg ICLF (*p* = 0.1023).

HR (Figure 5B and Table 1) increased during tilt (Tilt_10_, Tilt_20_, LBNP_30_ and presyncope time points) compared to baseline supine in the placebo and 0–60 mmHg ICLF conditions (*p* < 0.03). With 0–30 mmHg ICLF HR was increased compared to baseline at Tilt_10_, LBNP_30_ and presyncope time points (*p* < 0.01), but was not significant at Tilt_20_ (*p* = 0.1298). Throughout tilt, HR remained significantly lower in the 0–60 mmHg ICLF condition, compared to both placebo (*p* < 0.0001) and 0–30 mmHg ICLF (*p* = 0.0005). Furthermore, HR remained lower with 0–30 mmHg ICLF compared to placebo (*p* = 0.0404). When expressed as a percent change from baseline, the magnitude of the HR increase was significantly blunted with 0–60 mmHg ICLF relative to placebo (*p* < 0.0001) and 0–30 mmHg ICLF (*p* = 0.0005) and tended to be reduced with 0–30 mmHg ICLF compared to placebo (*p* = 0.0512). At presyncope, the magnitude of the HR increase (%) was significantly greater compared to Tilt_10_ and Tilt_20_ time points in all three conditions (*p* < 0.0001), but was only significantly greater than LBNP_30_ in the 0–60 mmHg compression condition (*p* < 0.0001).

In all three compression conditions, SV (Figure 6A and Table 1) was significantly reduced during tilt (Tilt_10_, Tilt_20_, LBNP_30_, and presyncope) compared to baseline supine (*p* ≤ 0.0003). SV remained significantly higher with 0–60 mmHg compression compared to placebo (*p* < 0.0001) and 0–30 mmHg ICLF (*p* < 0.0001). When expressed as a percent change from baseline, the magnitude of the SV reduction associated with tilt was decreased with 0–60 mmHg ICLF relative to placebo (*p* = 0.0001) and 0–30 mmHg ICLF (*p* = 0.0001).

During tilt (Tilt_10_, Tilt_20_, and LBNP_30_), CO (Figure 6B and Table 1) was not different from baseline supine, irrespective of condition. Overall, CO was higher in the 0–60 mmHg compression condition compared to 0–30 mmHg (*p* < 0.0001) and placebo (*p* < 0.0001), and was increased in placebo compared to 0–30 mmHg ICLF (*p* = 0.0074). However, when expressed as a percent change from baseline, CO was not different between conditions. At presyncope CO was reduced compared to baseline, Tilt_10_ and Tilt_20_ time points in all three conditions (*p* < 0.0028), but was only also reduced compared to LBNP_30_ in the placebo and 0–60 mmHg conditions (*p* < 0.0013).

During tilt (Tilt_10_, Tilt_20_, and LBNP_30_) and at presyncope, TPR (Table 1) was not different from baseline supine, irrespective of condition. Overall, TPR (absolute value or percent change from baseline) was increased in the 0–30 mmHg compression condition compared to both placebo (*p* ≤ 0.0300) and 0–60 mmHg ICLF (*p* ≤ 0.0125).

FVR (Table 1; reported only as percent change from baseline as noted previously) was not different between compression conditions throughout tilt. However, at presyncope, FVR was significantly greater in the 0–60 mmHg ICLF condition compared to placebo (*p* = 0.009) and 0–30 mmHg ICLF (*p* = 0.002), perhaps again reflecting that the test was stopped in some individuals in the 0–60 mmHg ICLF condition due to orthostatic symptoms or HR criteria, rather than presyncope.

For all conditions, CBFv (Figure 6C and Table 1) decreased during tilt (Tilt_10_, Tilt_20_, and LBNP_30_; *p* < 0.0001) and at presyncope (*p* < 0.0001) relative to baseline. Throughout tilt, CBFv (absolute value or percent change from baseline) remained higher with 0–60 mmHg ICLF compared to both 0–30 mmHg ICLF (*p* < 0.0001) and placebo (*p* < 0.0001).

Throughout tilt, P_ET_CO_2_ (Figure 6D and Table 1) was significantly increased with 0–60 mmHg compression compared to both placebo (*p* < 0.0001) and 0–30 mmHg ICLF (*p* < 0.0001). At presyncope, P_ET_CO_2_ was significantly reduced compared to Tilt_10_ and Tilt_20_ values for all conditions (*p* ≤ 0.0001), but was not different from LBNP_30_.

### Initial Systolic Arterial Pressure Responses

**FIGURE 7:**
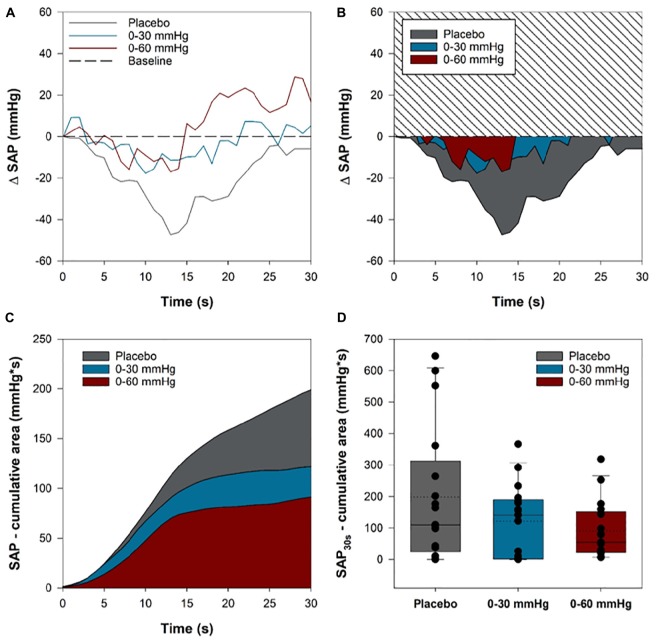
Systolic arterial pressure (SAP) reduction in the initial 30 s of head-up tilt. For each participant, the area of the SAP curve that dipped below baseline values throughout the first 30 s of HUT was integrated for each condition. The area under the curve of the SAP dip for was summed for each participant in each condition. **(A)** A sample trace from one participant showing the SAP time course in the first 30 s of tilt in the three conditions. **(B)** The integrated area between the baseline and SAP curves in each of the three conditions for the same sample participant. **(C)** The cumulative summed area for all participants in each of the three compression conditions throughout the first 30 s of tilt. **(D)** Boxplots comparing the cumulative summed area between the baseline and SAP curves for each participant in the three conditions. Solid lines show the sample median, while dashed lines depict the sample mean.

We evaluated the effect of intermittent compression on SAP throughout the initial 30 s of tilt to examine whether this intervention might be of benefit for the prevention of initial orthostatic hypotension (OH). For each participant we expressed SAP as an absolute change from baseline values (Figure 7A), and subsequently integrated the area of the SAP curve that fell below baseline during the initial 30 s of tilt for each condition (Figure 7B). Only participants with complete data from all three tilts were included in the analysis (*n* = 17). The cumulative sum of the integrated area for all participants throughout the first 30 s is shown in Figure 7C, and illustrates that ICLF, particularly 0–60 mmHg ICLF, tends to blunt the initial blood pressure drop associated with upright tilt. We compared the cumulative summed area of the SAP decrement between conditions using a linear mixed model analysis (Figure 7D), which showed that 0–60 mmHg ICLF tends to reduce the SAP drop compared to placebo (*p* = 0.052), whereas 0–30 mmHg ICLF is not sufficient to blunt the initial orthostatic SAP drop (*p* = 0.203). Three participants met criteria for initial OH (SAP decrease ≥ 40 mmHg or DAP decrease ≥ 2 0 mmHg within 15 s of standing) in the placebo condition, while one participant met criteria in the 0–60 mmHg condition, and none met the criteria in the 0–30 mmHg condition.

## Discussion

In this study, we demonstrated that intermittent calf compression from 0 to 60 mmHg (4 s on – 11 s off) effectively delays the onset of presyncope in healthy individuals. Our 0–60 mmHg ICLF paradigm successfully prevented venous pooling and capillary filtration in the calf, even reducing fluid accumulation relative to supine baseline (Kitayama et al., 2017; Tran and Argáez, 2017). This fluid was instead mobilized back into the effective circulating volume, perhaps with some enhancement in lymphatic drainage, improving SV, allowing blood pressure to be maintained at a reduced HR, and thus increasing cardiovascular autonomic reserve, improving CBFv, and delaying presyncope. The 0–60 mmHg ICLF outperformed both placebo and 0–30 mmHg ICLF in these respects. In this study, a smaller proportion of tilts were terminated due to presyncopal blood pressures in the 0–60 mmHg ICLF condition than with placebo or 0–30 mmHg ICLF. These tilts were instead terminated at participant request due to orthostatic symptoms at these high levels of orthostatic stress or due to orthostatic tachycardia; therefore, it is possible that the mean improvement in orthostatic tolerance of +7.4 min we observed with 0–60 mmHg ICLF may underestimate the true benefit of this intervention had these tests been continued until a presyncopal end point was reached in all participants. Regardless, an improvement in orthostatic tolerance of this magnitude, during profound orthostatic stress (tilting combined with LBNP) is a large and clinically meaningful improvement in orthostatic tolerance, comparable to that of salt supplementation, water ingestion and head-upright sleeping (Cooper and Hainsworth, 2002, 2008; Schroeder et al., 2002; Lu et al., 2003; Claydon and Hainsworth, 2004; Claydon et al., 2006).

We chose to also test ICLF at the level of 0–30 mmHg to examine whether there would be a dose-dependent effect on orthostatic tolerance, and also because if a lower pressure offers benefit, this would simplify device engineering. Our previous studies showed that pressures lower than 60 mmHg were sufficient to blunt pooling in the calf, but did not convey a measurable cardiovascular benefit during a short (10 min) orthostatic challenge (Hockin et al., 2019). We wondered whether reduced pooling would be sufficient to delay syncope in the face of a more severe orthostatic stress. Although 0–30 mmHg ICLF did indeed blunt venous pooling and capillary filtration in the calf, this pressure was not sufficient to produce a meaningful hemodynamic benefit, similar to our previous studies, and did not improve orthostatic tolerance compared to placebo.

In addition to the improved SV and HR responses we observed during the test, 0–60 mmHg ICLF was also associated with increased CBFv and P_ET_CO_2_. During profound orthostatic stress, the body engages the respiratory muscle pump to enhance venous return, with individuals experiencing a drive to increase breathing rate and depth (Miller et al., 2005). During deep inspiration, decreased intrathoracic pressure is transmitted to the cardiac chambers and thoracic blood vessels, creating a pressure gradient that promotes right atrial filling and venous return. Intra-abdominal pressure increases simultaneously, compressing both the iliac and femoral veins, reducing retrograde flow and further supporting an increased venous return (Miller et al., 2005). One negative consequence of activation of the respiratory muscle pump is that the increased ventilation decreases circulating CO_2_. CO_2_ is a potent vasodilator in the cerebral circulation, so the reduction in circulating CO_2_ results in decreased CBFv (Norcliffe-Kaufmann et al., 2008). The improved CBFv we observed with 0–60 mmHg ICLF in the present study appears to be largely due to decreased activation of the respiratory muscle pump, as the reduction in P_ET_CO_2_ levels associated with tilting was blunted compared to placebo and 0–30 mmHg ICLF (Figure 6D and Table 1). This is further evidence that intermittent calf compression increases hemodynamic reserve during orthostatic stress.

For intermittent calf compression to work effectively, both 0 mmHg holding pressures and rapid inflation rates are key. A 0 mmHg holding pressure allows the veins to fill between pulses, which is necessary for the venous pump to work effectively, and further prevents the formation of edema distal to the site of compression (Rucinkski et al., 1991; Lurie et al., 2003). Rapid inflation rates optimize venous return by maximizing peak flow velocities (Morris et al., 2006).

We chose to evaluate the ICLF interventions using head-up tilting rather than motionless standing for several reasons: (1) it allows the level of muscle activation to be standardized across all conditions; (2) it permits calf circumference to be measured using strain gauge plethysmography without artifact due to postural sway; (3) head-up tilting is a more intense orthostatic stress than active standing, and when combined with LBNP enables the achievement of a presyncopal end-point in the majority of participants.

To evaluate venous pooling and capillary filtration, we measured calf circumference using strain gauge plethysmography; this approach is well established (Whitney, 1953; Gamble et al., 1993; Halliwill et al., 1999; Rosfors et al., 2014; Oeffinger et al., 2015). Upon assuming an upright posture, predictable fluid shifts are known to occur in the legs, with a rapid (∼2 min) pooling phase and a slower (>2 min) filtration phase (Brown and Hainsworth, 1999). Our calf circumference data suggest that venous pooling may have been slightly blunted by the presence of the compression cuff wrapped around the calf, as calf circumference in the rapid pooling phase (2 min) in the placebo condition are lower here than in previous studies (Brown and Hainsworth, 1999; Hockin et al., 2019). After 10 min of 60° HUT, accrued pooling and filtration has been shown to be ∼100 mL in each calf (Brown and Hainsworth, 1999) thus we expect by preventing venous pooling with 0–60 mmHg ICLF, that ∼200 mL of fluid was mobilized back into the effective circulating volume. We monitored gaiter circumference to ensure that our intervention did not trigger edema distal to the site of compression. In this respect, we regard that the 0 mmHg holding pressure is crucial.

We considered the impact baseline calf circumference or MCSA might have on venous pooling and filtration and on the efficacy of 0–60 mmHg ICLF. There were no significant relationships between baseline calf circumference or MCSA and measures of venous pooling and filtration, or the efficacy of the intermittent compression paradigm at 0–60 mmHg. Accordingly, these findings suggest that the efficacy of intermittent calf compression is not limited by anthropometric calf measures.

We also considered whether baseline orthostatic tolerance influenced the efficacy of 0–60 mmHg ICLF to delay the onset of presyncope. The mean improvement in orthostatic tolerance was dependent on the placebo condition orthostatic tolerance, such that individuals with a lower baseline orthostatic tolerance experienced a greater benefit with 0–60 mmHg ICLF. The caveat of this relationship is that a smaller proportion of tests in the 0–60 mmHg ICLF condition were terminated due to true presyncope, determined as a SAP < 80 mmHg, so the true improvement in orthostatic tolerance, particularly at the extreme levels of orthostatic stress tolerated in the 0–60 mmHg may be underestimated. Furthermore, it is far more difficult to improve orthostatic tolerance when baseline orthostatic tolerance is already in excess of the normal range (in the −40 or −60 mmHg phases of LBNP where the orthostatic stress is severe) – there may be a ceiling effect beyond which further improvements in already exceptional orthostatic tolerance are more limited. This does not limit the implications of this intervention – those with the poorest orthostatic tolerance would be the target for such a device, and are also those most able to exploit use of ICLF to improve their orthostatic cardiovascular control and delay the onset of presyncope during orthostatic stressors.

Despite testing this intervention in a healthy population, these results are encouraging for the management of recurrent syncope, and likely underestimate the benefit we would see in a patient population. Previous studies have shown that patients with syncope experience exacerbated venous pooling and capillary filtration, and have an increased cerebral reactivity to hypocapnia (Norcliffe-Kaufmann et al., 2008). With 0–60 mmHg ICLF, the prevention of pooling was robust, even with very high intensity orthostatic stress, supporting the notion that this pressure would also be sufficient to prevent pooling in patient populations. In patients with excessive pooling, the hemodynamic benefit would likely be increased, as a greater volume of fluid would be mobilized back into circulation. In this respect, the effect on orthostatic blood pressure control would presumably be greater than we observed here in healthy controls, who defend their blood pressure effectively during orthostasis. As previously mentioned, the fall in P_ET_CO_2_ associated with tilt was blunted with 0–60 mmHg ICLF due to reduced activation of the respiratory muscle pump; this would be beneficial in patients with an increased sensitivity to hypocapnia, increasing CBFv and reducing symptoms of presyncope. In light of these favorable results, future studies are recommended to confirm the utility of this intervention in patient populations.

The benefits of this intervention are not limited to patients with orthostatic syncope secondary to hypotension (vasovagal or OH); intermittent calf compression could also be an effective option for patients with orthostatic intolerance due to Postural Orthostatic Tachycardia Syndrome (POTS). POTS patients exhibit excessive HR responses during orthostasis, and experience considerable reductions in CBFv, which are largely due to postural hypocapnic hyperventilation (Novak et al., 1998; Del Pozzi et al., 2014). Patients report palpitations, fatigue, “brain fog,” and experience symptoms of presyncope while upright. Based on our results, intermittent calf compression would be expected to reduce sympathetically-mediated increases in HR and improve CBFv for these patients, reducing symptoms and improving quality of life. Furthermore, our analysis of initial SAP responses suggest that this intervention would be valuable in mitigating initial OH. We showed that 0–60 mmHg ICLF tends to blunt the initial decrease in SAP within the first 30 s of tilting. This is particularly important because in the present study we activated the compression upon tilting. It is possible that activation of the intermittent compression paradigm a few seconds prior to tilting would have provided further benefit.

Because the management of recurrent syncope is challenging, with limited benefit from pharmacological therapies, there is a need for novel therapeutic approaches. Consequently, other research groups are also exploring the utility of active compression. Moein et al. (2017) evaluated the efficacy of an active calf compression brace on orthostatic cardiovascular responses. The brace, when wrapped around the calf, exerted a baseline counterpressure of 15 mmHg, and utilized shape memory alloys that could be actuated to apply a pressure up to 27 mmHg (Moein et al., 2017). During tilting, actuation of the brace improved SV and HR responses (Moein et al., 2017). Okamoto et al. (2016) evaluated the efficacy of a servo-controlled inflatable abdominal binder for the reduction of splanchnic venous pooling during active standing in patients with autonomic failure. The binder could be inflated to a pressure of 40 mmHg during active standing and was as effective as Midodrine at improving upright BP and reducing orthostatic symptoms (Okamoto et al., 2016). These groups have shown that their compression garments improve hemodynamic responses during short bouts of orthostasis, but have not yet shown that they can delay the onset of presyncope. Both of these novel compression garments are active in the sense that they can be actuated during standing, when the compression is needed, to limit venous pooling, but are then static as the compression pressure applied is constant. The mechanism of intermittent calf compression differs from these garments, as the intermittent nature of the compression mimics the skeletal muscle pump to enhance venous return, rather than simply preventing venous pooling. We expect that intermittent compression of the calf may have a comfort advantage over actuated compression as intermittent compression promotes venous return, preventing stagnancy and an associated build up of metabolites.

With the success of our intermittent calf compression paradigm at delaying the onset of presyncope, we anticipate that this study will be instrumental in the design and production of a “smart” ambulatory intermittent compression device. We envision that this device would rapidly (<1 s) engage, applying 4 pulses at 0–60 mmHg of pressure per minute (4 s on – 11 s off). The device might utilize dielectric elastomer actuators (Pourazadi et al., 2015) or shape memory alloys (Moein and Menon, 2014; Moein et al., 2017) to enable intermittent compression, and would contain accelerometers, allowing compression to be activated during motionless standing and deactivated during ambulation (when the skeletal muscle pump helps offset hemodynamic deficits) or during sitting/supine postures (when orthostatic stress is negligible), incorporated into a comfortable and portable garment.

### Limitations

This experiment was conducted in a single-blind fashion; the investigator that terminated the test was unaware of the study condition during testing. Double-blind testing was not possible because participants would be able to feel the compression intervention applied. However, the participants were unable to identify the compression condition consistently, and were not informed of the expected effect of the intermittent compression intervention. Investigators were blinded to the experimental condition during data collection and analysis.

We did not measure SV and CO directly; continuous estimates of SV and CO were calculated using the Modelflow^TM^ algorithm (Wesseling et al., 1993; Jellema et al., 1996; Harms et al., 1999). While this may be subject to inaccuracies when reported on an individual basis, aggregate changes in SV and CO determined by Modelflow^TM^ in response to cardiovascular stressors have been validated previously (Harms et al., 1999; Pitt et al., 2004; Manen et al., 2015).

In this study, we tested the efficacy of intermittent calf compression in a cohort of young healthy controls free of known cardiovascular disease or impairments. Accordingly, for reasons mentioned in the discussion, we expect that these results may underestimate the benefit that 0–60 mmHg ICLF would have in patient populations who have low orthostatic tolerance, and thus would be poised to experience greater benefit from the device. Nonetheless, the baseline (placebo) orthostatic tolerance of our cohort is lower than population norms. The mean orthostatic tolerance for males in this study was 29.6 ± 3.0 min, while for females it was 21.2 ± 2.9 min; while normative values for orthostatic tolerance are 35 ± 1.4 min in males, and 29 ± 1.5 min females (Protheroe et al., 2013). As intermittent calf compression was effective in this cohort, we are optimistic that we would see similar results in patient populations.

We did not objectively measure physical fitness, but are aware that many of our participants were quite physically fit; increased muscle bulk and fitness promote angiogenesis which, in turn increases the pooling potential and reduces orthostatic tolerance (Harrison, 1986; Pinckard et al., 2019). Perhaps this explains why their OT was slightly lower than the normative values.

## Conclusion

Intermittent calf compression from 0 to 60 mmHg (4 s on – 11 s off) effectively delays the onset of presyncope in young, healthy adults. The improvement in orthostatic tolerance was associated with reductions in venous pooling and capillary filtration, lower heart rates, higher stroke volumes, higher cerebral blood flow velocity and blunting of orthostatic hypocapnia secondary to activation of the respiratory muscle pump. Intermittent calf compression is a promising novel intervention for the management of disorders of orthostatic intolerance. This research will be instrumental in the design and production of a “smart” device that would activate during motionless standing, and inactivate during ambulation or recumbence to maximize both physiological gain and patient comfort.

## Data Availability Statement

Due to legal and ethical restriction, data cannot be made publicly available. Data will be made available upon request. Additional published or public analyses would only be permitted with ethics approval for secondary data access. Requests for access to datasets should be made to the corresponding author.

## Ethics Statement

Ethical approval was obtained from the Simon Fraser University Research Ethics Board and experiments were conducted in accordance with the Declaration of Helsinki. All participants provided written informed consent.

## Author Contributions

Together BH and VC conceived and designed the study, collected the data, and interpreted the results. BH analyzed the data, created the figures, and drafted the manuscript. BH and VC edited the manuscript and approved the final version for submission.

## Conflict of Interest

The authors declare that the research was conducted in the absence of any commercial or financial relationships that could be construed as a potential conflict of interest.
